# Anterior Cruciate Ligament (ACL) Reconstruction and Extra-Articular Tenodesis in a Contralateral Above-Knee Amputee Following Complex Trauma: A Case Report

**DOI:** 10.5704/MOJ.2203.018

**Published:** 2022-03

**Authors:** RM Vijayaraj, M Balakumaran, AR Ahmad, GN Solayar

**Affiliations:** 1 Department of Orthopaedics, Hospital Tuanku Ja'afar, Seremban, Malaysia; 2 Department of Orthopedic Surgery, International Medical University, Seremban, Malaysia

**Keywords:** modified LeMaire, ACL reconstruction, above knee amputation, complex femoral fracture, rehabilitation

## Abstract

We report the outcome following arthroscopic ACL reconstruction combined with a Modified LeMaire procedure in a patient who underwent multiple surgeries following an open ipsilateral femoral fracture and an above knee amputation of the contralateral limb at the time of initial trauma. This case highlights the importance of achieving ligamental stability in the contra-lateral limb in aiding proper rehabilitation following amputation and the potential pitfalls of retrograde femoral nailing.

## Introduction

Anterior cruciate ligament (ACL) injury in the contralateral knee following below- or above- knee amputation significantly complicates a patient’s rehabilitation. A stable contralateral knee is required due to the increased load stresses and gait abnormalities during the process of prosthetic fitting following amputation. The addition of an extra-articular tenodesis procedure during the time of ACL reconstruction may improve rotatory instability and overall outcomes compared to ACL reconstruction alone^1^.

In this article, we report our outcomes following an ACL reconstruction and a Modified LeMaire procedure in a patient who previously underwent a contra-lateral above knee amputation and ipsilateral femoral fracture fixation following trauma.

## Case Report

Our patient is a 27-year-old Malay male who suffered a severe motor vehicle accident in early 2016. At that time, he sustained multiple injuries including bilateral open femur fractures with the left femur associated with vascular compromise. He underwent an above-knee amputation of the left femur while the right femur was treated with an external fixator. After six months, the right femur fracture was fixed with a retrograde femoral nail for delayed union (he had undergone multiple debridement procedures due to complications of wound healing) and the fracture subsequently united.

**Fig. 1:**
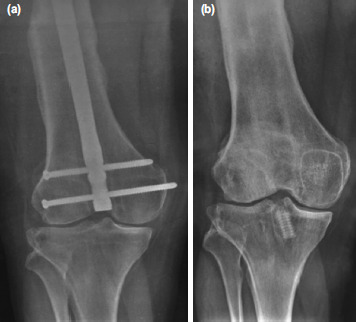
(a) Pre-operative showing the retrograde femoral nail and healed femoral fracture. (b) Post-operative following ACL reconstruction and Modified LeMaire.

Despite rehabilitation for over a year following union of the right femoral fracture, the patient was persistently unable to weight bear on his right lower limb due to knee pain and instability (Fig. 1a). Clinically, the patient exhibited a high-grade pivot shift on examination. The retrograde femoral nail was removed, and an MRI confirmed a complete ACL tear. As a result, prosthetic fitting was delayed, and the patient remained on wheelchair ambulation. Following repeated counselling of the patient and his family, they agreed for surgical stabilisation (Fig. 1b).

Due to financial constraints, surgery was delayed. During that time, our patient underwent regular sports physician rehabilitation with an above-knee prosthesis fitting of the left lower limb. In July 2019, patient underwent an arthroscopic right ACL reconstruction using an Achilles tendon allograft with a tenodesis screw fixation on both femur and tibia. Extra-articular tenodesis was performed through a separate incision using a modified LeMaire procedure.

**Fig. 2:**
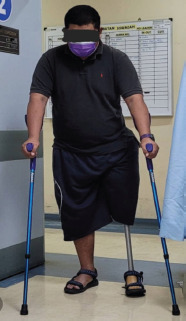
Our patient at his 18-month follow-up in the clinic (mobilising with crutches).

Post-operatively, the patient was placed in an extended knee brace initially and progressive knee flexion was allowed during subsequent follow-up by our sports physicians. Initial rehabilitation focused on quadriceps and hip abductor strengthening with a goal of establishing confidence in single leg stance. Once this was achieved, gait training using the contra-lateral prosthesis was commenced in a progressive manner. The prosthesis was custom made with a suction cup proximally and a single axis hinge (Hosmer Single Axis Friction Knee SAFK). The patient gradually regained knee range of motion with flexion beyond 100 degrees and was able to perform single-leg half-squats at three months postoperatively. The brace was discontinued at six months post-op and steady gait training with his prosthesis progressed well. At last follow-up (18 months post-surgery), the patient is now mobilising with one crutch, performing his activities of daily living independently, is pain free and very happy with his outcome thus far (Fig. 2).

## Discussion

The incidence of ipsilateral ACL injury following trauma necessitating contra-lateral lower limb amputation has been described before. In a study by Kilcoyne *et al*, the incidence of significant ligamentous injury in the opposite limb (following post-traumatic lower limb amputation) was 2% (8 out of 381 amputations). They noted that multi-ligamentous knee injuries are often unrecognised due to the distracting trauma involving the contralateral limb^2^. As our own case shows, it is important to maintain a high index of suspicion (due to the low incidence rate) as failure to recognise this would hinder proper rehabilitation post amputation.

Added to this, our patient suffered an ipsilateral open femoral fracture requiring multiple surgeries (external fixators; retrograde nailing). The incidence of ACL injuries following femoral shaft fractures has been reported to range between 27% and 57% and these are attributable to both the initial trauma and subsequent management of these fractures^3^. While there are pros and cons for both antegrade and retrograde femur nailing, there is evidence that retrograde nailing increases the risk of iatrogenic injury to the ACL, cartilage and menisci combined with poorer postoperative knee outcome scores^4^. In retrospect, antegrade nailing may have been the better option in preserving knee function and reducing the potential intra-articular damage to the knee for our case.

Post-operative functional outcomes in lower limb amputees are dependent on the success of an appropriate prosthesis combined with rehabilitation. To achieve this goal, the stability of the contralateral knee is crucial. In an amputee, joint forces in the contralateral limb exceed natural limits possibly due to the absence of functional locomotion and proprioceptive feedback from the amputated side^5^. We believe that the improved stability from an ipsilateral ACL reconstruction would be further enhanced with the addition of a Modified LeMaire extra-articular tenodesis which formed the rationale of our operative plan. While there are no specific studies that we are aware of on the use of the Modified LeMaire procedure in contralateral amputees, we postulate that the improved rotatory stability provided would improve the long-term outcome following ACL reconstruction in this case. As of last follow-up, the patient has progressed well with his rehabilitation and has no instability symptoms in his operated knee.

In conclusion, a successful outcome is possible following combined ACL reconstruction and a Modified LeMaire extra-articular tenodesis in a patient with significant ipsilateral femoral trauma and a contra-lateral above knee amputation. As far as we are aware, this is the first described case of utilising these methods in a complex case requiring above knee amputation. We also highlight the potential pitfall of performing retrograde femoral nailing in significant lower limb trauma where cruciate ligament injury is of concern (especially when considering a prosthesis following amputation.
